# Optimal variable identification for accurate detection of causal expression Quantitative Trait Loci with applications in heart-related diseases

**DOI:** 10.1016/j.csbj.2024.05.050

**Published:** 2024-06-03

**Authors:** Guishen Wang, Hangchen Zhang, Mengting Shao, Min Tian, Hui Feng, Qiaoling Li, Chen Cao

**Affiliations:** a College of Computer Science and Engineering, Changchun University of Technology, Changchun 130012, China; b Key Laboratory for Bio-Electromagnetic Environment and Advanced Medical Theranostics, School of Biomedical Engineering and Informatics, Nanjing Medical University, Nanjing 211166, China; c Department of Cardiology, Affiliated Drum Tower Hospital, Medical School of Nanjing University, Nanjing 210008, China

**Keywords:** Genome-wide association studies, Expression quantitative trait loci, Penalized regression, L0 regularized regression, Causal eQTL variants

## Abstract

Gene expression plays a pivotal role in various diseases, contributing significantly to their mechanisms. Most GWAS risk loci are in non-coding regions, potentially affecting disease risk by altering gene expression in specific tissues. This expression is notably tissue-specific, with genetic variants substantially influencing it. However, accurately detecting the expression Quantitative Trait Loci (eQTL) is challenging due to limited heritability in gene expression, extensive linkage disequilibrium (LD), and multiple causal variants. The single variant association approach in eQTL analysis is limited by its susceptibility to capture the combined effects of multiple variants, and a bias towards common variants, underscoring the need for a more robust method to accurately identify causal eQTL variants. To address this, we developed an algorithm, CausalEQTL, which integrates *L*_0_ +*L*_1_ penalized regression with an ensemble approach to localize eQTL, thereby enhancing prediction performance precisely. Our results demonstrate that CausalEQTL outperforms traditional models, including LASSO, Elastic Net, Ridge, in terms of power and overall performance. Furthermore, analysis of heart tissue data from the GTEx project revealed that eQTL sites identified by our algorithm provide deeper insights into heart-related tissue eQTL detection. This advancement in eQTL mapping promises to improve our understanding of the genetic basis of tissue-specific gene expression and its implications in disease. The source code and identified causal eQTLs for CausalEQTL are available on GitHub: https://github.com/zhc-moushang/CausalEQTL.

## Graphical Abstract

**Figure:**
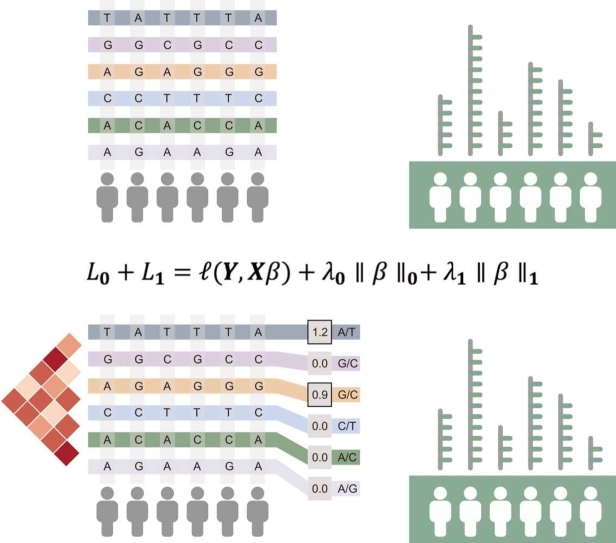

## 1 Introduction

Genome-wide association studies (GWAS) have been instrumental in uncovering genetic variations linked to a wide array of complex traits and diseases, significantly enhancing our understanding of the genetic underpinnings of various phenotypes[1]. Despite this progress, the genetic variants identified by GWAS often explain only a limited proportion of phenotypic variance. It is increasingly recognized that these variants may exert their effects through the modulation of gene expression, which is a key molecular phenotype[2]. In this context, transcriptome-wide association studies (TWAS) have emerged as a successful strategy, uncovering genes associated with a range of diseases, including cancer[3], [4], [5]. This approach is grounded in the hypothesis that genetic variants influence gene expression, which in turn affects the phenotype.

The eQTL studies systematically explore how genetic variations impact gene expression, offering a powerful tool to establish associations between genotype and expression phenotypes[6], [7]. Through eQTL analysis, we can not only discern the direct relationship between genotype and expression but also understand how these associations function under both physiological and pathological conditions[8], [9]. This understanding is vital for in-depth analysis of disease mechanisms, as eQTL serves as direct links between genetic variation and gene expression levels, aiding in the unraveling of gene regulatory networks and identifying potential therapeutic targets.

While studies like those by Farh et al.,[10] have identified single nucleotide polymorphisms (SNPs) linked to autoimmune diseases through GWAS, only about 12 % of these SNPs were predicted as eQTLs. This suggests that the majority of SNPs associated with autoimmune diseases may not directly regulate gene expression or have a minimal impact on it. Given the limitations of GWAS in explaining the functional consequences of genetic variation, eQTL studies have emerged as a crucial complementary approach. For instance, findings of Chun et al.,[11] indicate that some genetic loci associated with autoimmune and inflammatory diseases align with eQTL functioning in specific immune cell subtypes. However, these represent only a subset of the eQTLs in disease risk loci, underscoring the need for comprehensive exploration of eQTLs in these diseases.

This hypothesis gains relevance when a variant associated with a GWAS locus also impacts gene expression, potentially unveiling mechanisms of gene regulation and disease. However, confirming a variant's concurrent role in both GWAS and expression quantitative trait locus (eQTL) studies is fraught with challenges. These challenges stem from the complexities of linkage disequilibrium and the presence of multiple causal variants at some loci. The analysis of eQTL across various tissues is crucial for investigating both the tissue-specific characteristics of eQTLs and their broader impact on different tissue types[12]. In this regard, the GTEx project[13], which includes a wide range of tissues and individual data, provides an invaluable resource for calculating eQTLs in different tissues[14]. Through these efforts, tens of thousands of eQTLs, some tissue-specific, have been identified, largely through single-variant association analysis. This method involves testing multiple SNPs per gene independently, identifying the most significantly associated SNP, and using a permutation-adjusted *P*-value to control the overall false discovery rate (FDR).

However, this single-variant association approach has several drawbacks[15]. First, noncausal eQTL variants can appear most strongly associated with a gene due to linkage disequilibrium (LD). Second, it fails to estimate combined effects from multiple causal eQTL variants, which is problematic when two or more regulatory variants jointly affect gene expression. Third, common variants tend to have higher *P*-values than lower-frequency variants of equal effect size, despite the potential contribution of rare noncoding variants to individual gene expression levels. These rare variants are more likely to be deleterious than common variants, making it crucial to identify rare causal eQTL variants[16]. Therefore, a robust approach for identifying causal eQTL variants that overcomes these drawbacks of single-variant association analysis is highly desirable.

The widely used method for detecting the causal variant is fine-mapping, which aims to identify the causal variant from the significant signal in GWAS summary statistics[17]. The general strategy of fine-mapping involves selecting a region of interest from the GWAS list of SNPs associated with a trait. Subsequently, the LD structure and genes mapped to this region are visually explored, followed by an assessment of the likely function of selected SNPs based on publicly available genomic annotation. Finally, variants in the genomic region most likely to be causally related to a trait are determined after accounting for the correlation between variants in the region. Meanwhile, the power of fine-mapping is affected by multiple factors, such as the frequency of variants, the local LD structure, and sample size.

The challenges in this field arise from the complexities of linkage disequilibrium and the presence of multiple causal variants at some loci. Additionally, the sample sizes in resources like GTEx are limited, especially for certain tissues, thus reducing the power of traditional algorithms for eQTL calculation. Therefore, accurately locating eQTLs is of paramount importance. In eQTL analysis, linear models such as the Least Absolute Shrinkage and Selection Operator (LASSO)[18], Elastic Net[19], and Bayesian methods[20] are widely utilized. LASSO and Elastic Net, as types of regularization regression methods, introduce sparsity into model coefficients, aiding in selecting the most informative eQTLs and reducing the risk of overfitting. Specifically, LASSO, through *L*_1_ regularization, automatically selects features by shrinking many parameters to zero. *L*_1_ regularization (LASSO) is often considered the optimal convex approximation of *L*_0_ regularization. As the *L*_0_ norm is non-convex, its optimization problem is relatively challenging. Hazimeh et al., recently introduced an efficient framework, a package called L0Learn[21], using a coordinate descent algorithm to solve *L*_0_ regularization learning problems. Compared to popular sparse learning tools, this algorithm achieves competitive runtime and encouraging results for optimal feature selection. Our work focuses on enhancing the accuracy of detecting eQTLs by applying the *L*_0_ +*L*_1_ regularized regression, aiming to overcome the limitations of existing models and provide a more accurate algorithm for calculating causal eQTL.

## 2 Materials and methods

### 2.1 Dataset

In this study, we utilized data from the Genotype-Tissue Expression project (GTEx)[13] as our primary training dataset and testing dataset. GTEx, initiated by the National Institutes of Health (NIH) in the United States, aims to investigate tissue-specific gene expression and regulation in diverse human tissues. The dataset includes genetic data from 948 pre-mortem healthy human contributors and gene expression data from 17,382 RNA-seq samples, providing sufficient power to identify eQTL in 48 tissues. The GTEx dataset comprises RNA-seq and genotype data for 54 tissues, from which we have selected only those with sample sizes greater than 100. This research leverages the strengths of GTEx datasets to enhance our understanding of the genetic landscape across populations and gene expression across tissues. Specifically, we use GTEx whole blood samples, which include genotype data, as a simulation source for known gene expression. Various algorithms are then applied to assess their performance in accurately calculating eQTL.

### 2.2 Simulation procedure

In our analysis, we cataloged all SNPs from the GTEx datasets. We grouped SNPs associated with the same disease-related gene, resulting in a total of 24,326 genes (including 20,300 genes with two or more SNPs and 19,409 genes with three or more SNPs). For each gene, we randomly selected 2 and 3 SNPs as random eQTLs and then predicted the gene expression values for each gene using a gene expression simulation procedure below.

The simulation encompasses four genetic architectures: additive, heterogeneous, recessive, and compensatory interactions. In our predictive model, each gene is confined to a specific region, encompassing the gene's original location sequence. In the additive architecture, several SNPs are randomly chosen within this gene region. The contribution of these SNPs to gene expression is determined by coefficients drawn from a(0,1) Gaussian normal distribution. Therefore, the gene expression, prior to considering heritability, is the cumulative effect of these SNPs. We utilized specific genetic architectures to generate gene expression with a predetermined heritability ($h^{2}$). To achieve this, we first established the variance of the genetic component of the gene expression, denoted as $\sigma_{g}^{2}$. We then calculated the variance of the environmental component, $\sigma_{e}^{2}$, to ensure that the ratio of genetic variance to the total variance $\frac{\sigma_{g}^{2}}{\sigma_{g}^{2}+\sigma_{e}^{2}}=h^{2}$ equaled the preselected heritability value ($h^{2}$). Following this, we sampled from a normal distribution to quantify the influence of non-genetic factors, such as environmental effects or random noise, on the gene expression. For the other genetic architectures, the simulation of gene expression, which incorporates the predetermined heritability, follows a process similar to that of the additive architecture.

For the other three genetic architectures - heterogeneous, recessive, and compensatory interactions – the focus is on how SNPs contribute to gene expression without nonlinear effects. Detecting eQTLs in these scenarios and other nonlinear models can be more challenging, but nonlinear effects are often present. In a heterogeneous architecture, we randomly select two SNPs to examine the impact of genetic variation on gene expression. Here, individuals carrying alternative alleles at these SNPs will exhibit changes in gene expression, regardless of whether they carry one or both alternative alleles. In the recessive model, a change in gene expression occurs only when both alternative alleles are present. Conversely, in the compensatory model, gene expression changes only if exactly one alternative allele of the two SNPs is present.

### 2.3 CausalEQTL algorithm

LASSO (*L*_1_ regularization), as noted in works by Albanese and Donati[22] and Leviyang et al., [23], has been extensively applied in gene expression prediction and TWAS[24]. However, LASSO encounters limitations with SNPs in high linkage disequilibrium (LD), struggling to accurately identify causal variants due to minimal differences among these LD variants. This issue stems from *L*_1_ regularization penalizing the sum of the absolute values of regression coefficients, aligning the sum of SNP frequencies more closely with gene expression. Consequently, LASSO fails to differentiate between outcomes involving a few impactful SNPs and those with many SNPs of minor frequencies, leading to inconsistencies in identifying limited causal variants. Essentially, *L*_1_ does not yield sparse solutions when SNP differences are small, a common scenario in LD.

To enhance parsimony and minimize the identification of non-causal variants, we integrate *L*_0_ regularization, further refining the selection of SNPs. This addition underscores the necessity of *L*_0_ regularization in our approach, despite its slower processing speed. The recent development of the L0Learn package[25], [26], [27] marks a significant advancement in computer science for *L*_0_-based optimization. L0Learn is an approximate algorithm designed to solve *L*_*0*_ regularization problems and obtain high-quality solutions. The algorithm combines coordinate descent (CD) with local combinatorial optimization techniques to effectively tackle sparse learning problems[28], [29]. However, the standard cyclic coordinate descent algorithm's convergence results do not apply to cases with discontinuous objective functions[30]. Therefore, they proposed an improved variant of cyclic coordinate descent for converging to the local minimum value. The algorithm operates by first utilizing local search techniques to discover better solutions within the local neighborhood. Then, this new solution is used as an initial solution for further optimization using the coordinate descent algorithm. By iteratively applying these two methods, L0Learn is capable of quickly and effectively finding high-quality solutions. The advantage of this approach lies in its integration of the strengths of both local search and coordinate descent algorithms, enabling the algorithm to achieve better performance in handling sparse learning problems. This innovation greatly enhances the capability of CausalEQTL to perform this sophisticated inference.

(1) $$\begin{array}{c} J\left(\beta \right)=l(Y,\;X\beta )+\lambda_{0}∥\beta {∥}_{0}+\lambda_{1}∥\beta {∥}_{1}\# \end{array}$$

**Fig. 1:**
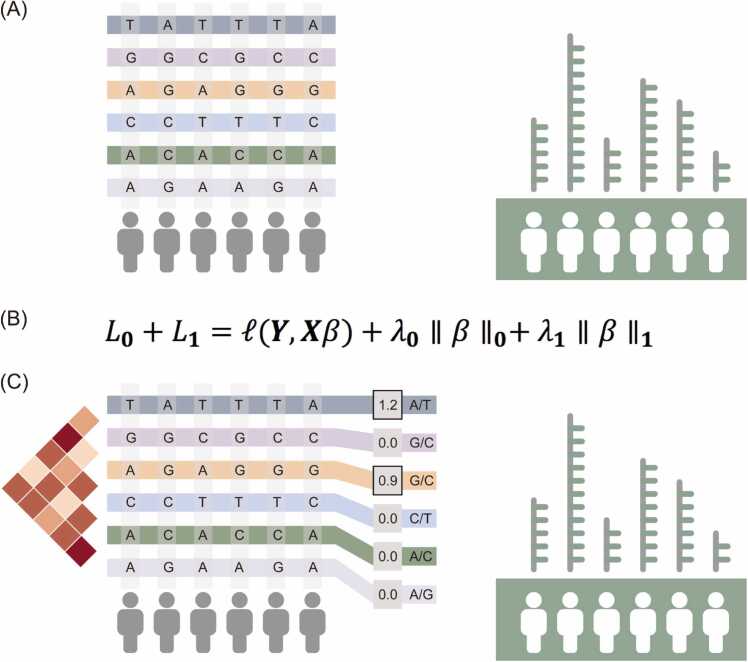
CausalEQTL Algorithm Protocol. (A) The primary objective of CausalEQTL is to identify causal eQTLs amidst SNPs with high linkage disequilibrium in gene regions. (B) The object in the *L*_0_ +*L*_1_ strategy of CausalEQTL employs *L*_0_ regularization to enhance feature selection sparsity, effectively discarding high LD SNPs. *L*_1_ regularization ensures that the predicted gene expression aligns with the sequenced transcriptome. (C) In the output of CausalEQTL, SNPs assigned with weights are identified as the final causal eQTLs by the CausalEQTL.

In Eq. (1), l is the loss function, it is defined using squared error loss in analysis. ***Y*** is the predicted expression value vector, ***X*** is the SNPs matrix, ***β*** is the coefficient matrix of the model. This ***X*** is two-dimensional, where one represents the variants and another represents individuals (Fig. 1). The term $\lambda_{0}∥\beta {∥}_{0}$represents the *L*_0_ regularization parameter, which governs the number of non-zero elements in the parameter vector. $\lambda_{1}∥\beta {∥}_{1}$ is the *L*_1_ regularization parameter, employed to encourage sparsity in the parameter vector.

### 2.4 Evaluation metrics

To validate the superiority of our method, we conducted a comparative analysis with six different methods: CaVEMaN[31], LASSO, Elastic Net, Ridge, DAP-G[32], and TensorQTL[33]. TensorQTL is a TensorFlow-based FastQTL, utilizing pandas-plink for the efficient importation of genotypes in PLINK format into dask arrays. Each gene was trained individually to determine the results. A non-zero value indicates that the model has identified the SNP as a weighted, causal eQTL feature. Conversely, a zero value implies that the SNP was disregarded by the model. For Ridge, all SNP weights are inherently non-zero. Therefore, we set a specific threshold (0.6); weights below this threshold are considered unselected, while those above are deemed selected. Despite the relatively high threshold for Ridge, we observed that the number of weighted SNPs it calculated significantly exceeded our simulation settings.

In the case of TensorQTL, the results are presented as *P*-values. We established a similar threshold to determine significance, set at 0.05 divided by the number of SNPs in the gene, to control for Type I error. A *P*-value below this threshold indicates that the feature is selected by the model, thus identifying it as a selected eQTL. If the *P*-value is above the threshold, the feature is considered unselected. This meticulous approach enabled us to thoroughly evaluate and compare the efficacy of each regression model and mapping mode in our study.

The F1 Score is a statistical metric used to evaluate the performance of a binary classification model. It is a comprehensive measure that considers both the precision and recall of the model. The F1 Score is calculated as the harmonic mean of precision and recall, used in the comparison in this study, defined as follows:

(2) $$\begin{array}{c} F1=\frac{2\times \mathrm{Precision}\times \mathrm{Recall}}{\mathrm{Precision}+\mathrm{Recall}} \end{array}$$

(3) $$\begin{array}{c} \mathrm{Precision}=\frac{\mathrm{TP}}{\mathrm{TP}+\mathrm{FP}} \end{array}$$

(4) $$\begin{array}{c} \mathrm{Recall}=\frac{\mathrm{TP}}{\mathrm{TP}+\mathrm{FN}} \end{array}$$

In these formulas, Precision represents the ratio of correctly predicted positive observations to the total predicted positives, as shown in Eq. (2). Recall, as described in Eq. (3), indicates the ratio of correctly predicted positive observations to all actual positives. Here, TP (True Positive) refers to instances where the model correctly identifies a positive sample. FP (False Positive) denotes cases where the model incorrectly labels a negative sample as positive. FN (False Negative) occurs when the model incorrectly classifies a positive sample as negative. In our studies, the F1 Score is utilized to measure the consistency of the SNPs selected by different models with the simulated causal SNPs. This application of the F1 Score helps in assessing the accuracy of our models in identifying SNPs that are truly influential in the studied genetic context.

## 3 Results

### 3.1 Overview of CausalEQTL

CausalEQTL employs an *L*_0_ +*L*_1_ penalized regression approach to identify SNPs with co-effects, which are considered causal eQTLs (Fig. 1). In our simulations, we explore various genetic architectures, including both linear and non-linear models. While linear architectures offer mathematical simplicity, they often fall short in capturing the complex biological processes where non-linear SNP interactions significantly influence gene expression changes, a very common scenario in transcriptomics[34], [35]. Our algorithm addresses this by incorporating three non-linear architectures in simulations, providing a more comprehensive analysis.

To validate the performance of CausalEQTL, we conducted tests using both simulated and real data across various genetic heritability scenarios. Our findings indicate that CausalEQTL consistently outperforms traditional linear models and the single-variant association approach (FastQTL[36]). Specifically, when applied to real GTEx data encompassing 48 tissue types, CausalEQTL effectively calculated eQTLs for each tissue and revealed a high degree of similarity in causal eQTL variants between closely related tissues. These results not only validate the effectiveness of CausalEQTL but also highlight its capability to discern tissue-specific genetic influences, underscoring the algorithm's robustness and accuracy.

### 3.2 Evaluating the performance of CausalEQTL on simulated data and its comparison with other algorithms

To validate the performance of CausalEQTL, we conducted tests using both simulated and real data across various genetic heritability scenarios. Our findings indicate that CausalEQTL consistently outperforms traditional linear models and the single-variant association approach (TensorQTL[33]).

**Fig. 2:**
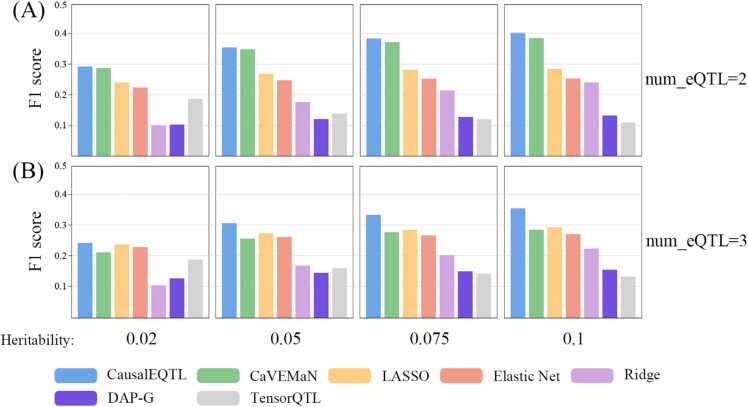
F1 Score Comparison between CausalEQTL and other algorithms for additive models. The algorithms compared include CaVEMaN, LASSO, Elastic Net, Ridge, DAP-G, and TensorQTL. (A) F1 scores when the number of true causal eQTLs is set to 2. (B) F1 scores when the number of true causal eQTLs is set to 3.

The comparative results for the simulated additive model are depicted in Fig. 2. Taking an example where the number of random eQTLs is set to 2 and the heritability is 0.02, the F1 scores are as follows: CausalEQTL at 0.29, CaVEMaN at 0.29, LASSO at 0.24, Elastic Net at 0.23, Ridge at 0.10, DAP-G at 0.10, and TensorQTL at 0.19 (Fig. 2A). Notably, CausalEQTL outperforms LASSO by 20 %, Elastic Net by 26 %, and TensorQTL by 53 %. Additionally, CausalEQTL selected 37,755 SNPs, compared to 89,759 SNPs by LASSO and 129,240 SNPs by Elastic Net. This demonstrates that CausalEQTL method not only leads in F1 score but also selects a limited number of SNPs, significantly reducing the occurrence of false-positive eQTLs and thereby accelerating subsequent analyses. CausalEQTL method is close to the results of CaVEMaN. We speculate that the reason is that CaVEMaN found only one eQTLs on most genes. This improves its F1 score with only 2 random eQTLs on each gene. When the heritability increases from 0.05 to 0.1, the F1 score of CausalEQTL is 1.4 %, 3.1 % and 4.6 % higher than CaVEMaN respectively. As heritability increases, the F1 score generally improves for most algorithms. Consider an example where the number of random eQTLs is set to 2. At a heritability of 0.05, CausalEQTL shows a 21 % increase, CaVEMaN a 21 % increase, LASSO a 12 % increase, Elastic Net a 10 % increase, Ridge a significant 76 % increase, and DAP-G a 17 % increase. However, TensorQTL's performance decreases. This decline is attributed to TensorQTL selecting more SNPs that are in linkage disequilibrium (LD) with the selected eQTLs, consequently lowering its F1 score. Additionally, when the number of random eQTLs rises from 2 to 3, a decrease in performance is observed across all methods (Fig. 2B). Specifically, at a heritability of 0.02, our CausalEQTL shows a more pronounced decrease but still maintains superiority over the other methods.

**Fig. 3:**
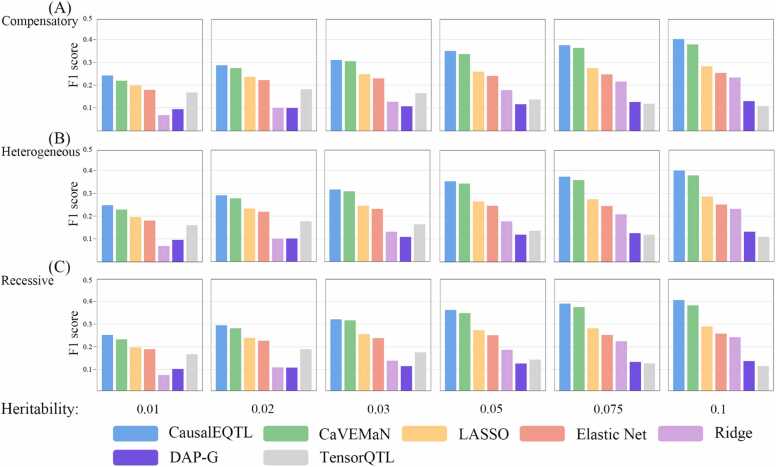
F1 Score comparison for non-additive architectures using CausalEQTL and other algorithms. The algorithms compared include CaVEMaN, LASSO, Elastic Net, Ridge, DAP-G, and TensorQTL. (A) F1 scores for the compensatory architecture. (B) F1 scores for the heterogeneous architecture. (C) F1 scores for the recessive architecture.

In this section, we present simulation results for three genetic architectures: compensatory, heterogeneous, and recessive, with the number of random eQTLs set to 2. The outcomes are illustrated in Fig. 3. For instance, Fig. 3A focuses on a compensatory genetic structure, and the second panel of Fig. 3B, depicting a heterogeneous genetic structure with a heritability of 0.02, shows the F1 score of CausalEQTL as 0.29, CaVEMaN at 0.28, LASSO at 0.24, Elastic Net at 0.23, Ridge at 0.10, DAP-G at 0.10, and TensorQTL at 0.18, while the Fig. 3C addresses a recessive architecture. We also analyze the comparative trends for each genetic architecture. Generally, most algorithms exhibit an increase in F1 score with higher heritability, particularly noticeable in our algorithm, CaVEMaN, and LASSO. However, for TensorQTL, as heritability increases, there is a notable decrease in the F1 score. This trend aligns with the observations for the simulated additive model discussed in Fig. 2, suggesting a consistent pattern across different genetic structures. As the number of designated eQTLs increases, the F1 score for all algorithms decreases, indicating that a greater number of eQTLs introduces more complexity into the computational process. These results demonstrate that CausalEQTL not only achieves a higher F1 score in additive architecture but also maintains robust performance when applied to non-linear genetic architectures.

Specifically, when applied to real GTEx data encompassing 48 tissue types, CausalEQTL effectively calculated eQTLs for each tissue. Notably, our analysis revealed a high degree of similarity in causal eQTL variants between closely related tissues, such as the artery aorta and artery tibial, with F1 scores reaching as high as 0.57. In contrast, distinct tissues showed significantly less overlap, such as the lung and uterus, with F1 scores reaching as low as 0.3. These results not only validate the effectiveness of CausalEQTL but also highlight its capability to discern tissue-specific genetic influences, underscoring the algorithm's robustness and accuracy.

In order to prove the effectiveness of CausalEQTL on real expression data. We selected whole blood tissue with the largest number of samples for calculation. After preprocessing, there were a total of 6070 genes. The accuracy rates of the 6 methods are as Table 1, more relevant information can be found in supplementary data (Table S1–3).

**Table 1:** Accuracy of each method.

Methods	CausalEQTL	CaVEMaN	LASSO	Elastic Net	DAP-G	TensorQTL
Accuracy	48.9 %	69.5 %	43.8 %	46.7 %	48.7 %	90.3 %

We acknowledge the performance disparity between our method, CausalEQTL, and CaVEMaN, TensorQTL. This discrepancy arises primarily from the distinct approach to eQTL calculation. While traditional methods focus on individual SNPs in eQTL calculations, CausalEQTL adopts the assumption of transcriptome-wide association study (TWAS), considering the contribution of multiple SNPs to gene expression as either additive or non-additive models, rather than just single SNPs. TWAS has proven to be highly effective in identifying numerous complex disease susceptibility genes[37]. Consequently, when benchmarked against the GTEx eQTL database, CausalEQTL exhibits inferior performance compared to CaVEMaN and TensorQTL. However, when evaluated under the TWAS assumption, CausalEQTL demonstrates superior performance. Furthermore, our utilization of the *L*_*0*_+*L*_*1*_ strategy represents a novel approach to eQTL detection, marking the first instance of applying this strategy under the assumption of additive eQTL contributions to gene expression.

### 3.3 Evaluating the performance of CausalEQTL on heart-related disease

**Fig. 4:**
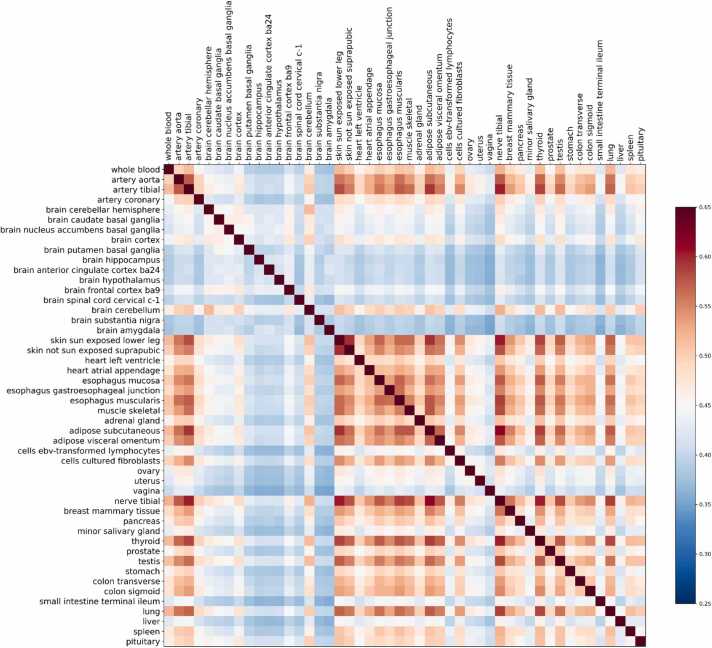
Heatmap of causal eQTL similarity across 49 GTEx tissues The heatmap color gradient represents the similarity between two tissues based on causal eQTL comparisons.

In our study, we explored tissue specificity patterns and mechanisms in eQTL analysis by quantifying the pairwise similarity of GTEx tissues using F1 scores derived from eQTL analysis results. Notably, the highest correlation was observed between artery aorta and artery tibial tissues, while the lowest was between vagina and brain amygdala (Fig. 4). Tissues sharing similar characteristics exhibited higher correlations. For instance, artery tissues from the aorta and tibia exhibited a notable correlation, with F1 score reaching 0.57, similar to the correlations found in adipose and skin tissue pairs, all of them exceed the overall mean F1 score of 0.45. These results underscore our method's efficacy in discerning relationships between related tissues. However, brain-related tissues demonstrated lower similarity patterns compared to the average, likely influenced by their smaller sample sizes. The average sample size for brain tissues was 167, below the GTEx average of 282. Additionally, the lower correlation between brain-related tissues and other non-brain tissues aligns with findings from previous studies[38]. These observations affirm the robustness of our tool while also highlighting the impact of sample size on its power. CausalEQTL may not achieve high accuracy in tissues with limited sample sizes, possibly due to insufficient heritability of gene expression relative to the available sample size.

The effects of different alleles of each eQTL on the expression of eGENEs in heart tissue (including the left ventricle and atrial appendage) were calculated using ANOVA analysis. Out of 45,368 pairs observed, 9412 showed a significant effect (ANOVA *P*-value < 0.05) in the atrial appendage tissue, while in the left ventricle tissue, 9455 out of 47,136 pairs exhibited significant relationships. The rationale for selecting heart tissue is its sufficient sample size to represent the average sample size of GTEx datasets. These results indicate that the eQTLs identified by CausalEQTL affect gene expression to a certain extent in heart tissue.

**Fig. 5:**
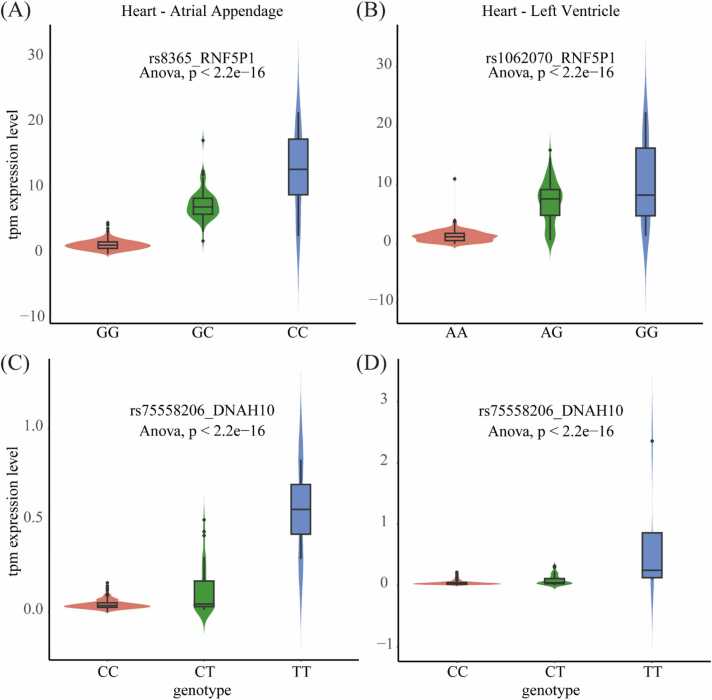
The effect of eQTL allele on the expression of eGENE in heart tissue (A) The impact of rs8365 on the expression of RNF5P1 in atrial appendage tissue. (B) The impact of rs1062070 on the expression of RNF5P1 in left ventricle tissue. The effect of rs75558206 on the expression of DNAH10 (C) in atrial appendage tissue and (D) in left ventricle tissue.

For further analysis, a comprehensive literature review was conducted. Yang et al. [39] and Ma et al. [40] identified lncRNA RNF5P1 as a novel biomarker for ischemic stroke. Higher expression of RNF5P1 correlates with an increased risk of ischemic stroke, highlighting its potential role in stroke pathogenesis. CausalEQTL identified rs8356 as the eQTL of RNF5P1 in heart atrial appendage tissue (Fig. 5A) and rs1062070 in heart left ventricle tissue (Fig. 5B). rs8365 is a single nucleotide variation located at position 32,148,403 on chromosome 6 (GRCh37), inducing a G to C mutation. The mutated allele significantly increases the expression of RNF5P1 (ANOVA *P*-value < 0.05) (Fig. 5A). Similarly, the mutated allele of rs1062070, a single nucleotide variation located at position 32,148,031 on chromosome 6, significantly increases the expression of RNF5P1 in the left ventricle tissue (Fig. 5B). RNF5P1 is a pseudogene of ring finger protein 5 (RNF5). As an E3 ubiquitin ligase, RNF5 catalyzes protein ubiquitination and plays an important role in cell proliferation, apoptosis, DNA damage repair, and immune response[41], [42]. The occurrence and development of ischemic stroke are accompanied by the inflammatory process[43], [44]. Additionally, Liu Chunjie et al. [45] identified DNAH10 as a novel candidate gene in patients with heterotaxy syndrome and congenital heart defects. In the DisGeNET database[46], DNAH10 is associated with atrial fibrillation with a score of 0.4. CausalEQTL identified rs75558206 as an eQTL of DNAH10 in both heart atrial appendage tissue and heart left ventricle tissue (Fig. 5C, D). The mutated allele of rs75558206 significantly increases the expression of DNAH10 (ANOVA *P*-value < 0.05). These results indicate that CausalEQTL is beneficial in discovering eQTLs of disease-related genes.

## 4 Discussion and conclusion

L0Learn[21], [25], [27] has gained widespread recognition in bioinformatics research, with successful applications in haplotype reconstruction[47], [48], polygenic score (PGS)[49], [50] calculation, and significant signal selection[51], et. al.[52] The key to L0Learn's success lies in its ability to produce sparse solutions in feature selection, particularly for similar features. LASSO contributes by accumulating the gene expression contributions of SNPs, aligning them more closely with sequenced gene expression data. Consequently, the *L*_0_ +*L*_1_ algorithm used in this study, despite its simplicity, achieves remarkable results in scanning for causal eQTLs. When compared to other linear models and single-variant association approaches, CausalEQTL demonstrates superior accuracy in pinpointing causal eQTLs, especially in the context of non-linear SNP interactions.

We also analyzed eQTLs calculated by our algorithm across all tissues in the GTEx database. The results reveal that tissues with closer biological relationships share a higher similarity in eQTLs, further validating the effectiveness of CausalEQTL. Similar eQTL patterns observed in closely related tissues within the GTEx dataset suggest that these tissues share analogous gene expression regulatory mechanisms. Thus, this not only underscores the algorithm's precision but also its capability to discern tissue-specific genetic influences, reinforcing its utility in complex genomic studies.

The CausalEQTL algorithm, proposed based on *L*_*0*_*+L*_*1*_ regularized regression, aims to identify causal eQTL across diverse conditions. It holds applicability across a wide spectrum of diseases and traits. Nevertheless, the statistical power of CausalEQTL may be limited by varying genetic architectures and regulatory mechanisms inherent to different traits and populations. We acknowledge that a minimum number of samples is required for effective analysis. Upon examining the correlation of eQTLs across all GTEx tissues, we observed that similar tissues tend to exhibit similar eQTLs. For instance, the F1 score for two closely related skin tissues is as high as 0.596. However, in the case of brain-related tissues, the similarity in eQTLs identified by our algorithm is not as pronounced. This discrepancy can be attributed to the smaller sample sizes available for GTEx brain tissues, highlighting a limitation of our current algorithm. Future improvements could include developing an algorithm specifically tailored to perform robustly with smaller sample sizes and integrating multiple cohorts representing diverse populations to illuminate the algorithm's performance and generalizability across varied contexts.

Transcriptome-wide association studies (TWAS) represent a strategy for associating 'imputed' gene expression with phenotypes[53], [54]. More than two dozen linear models have been employed in the development of TWAS algorithms, with approaches like Elastic Net (PrediXcan[55]) and Bayesian linear models such as BSLMM (FUSION[24]) demonstrating high accuracy. However, Bayesian models assume that all SNPs contribute to gene expression with small effects, which may not always be the case. While some studies, such as sTF-TWAS[56], have focused on transcription factors, there remains a gap in specifically addressing and utilizing 'true' causal eQTLs for TWAS. Moving forward, we aim to develop a TWAS strategy that centers on 'real' causal eQTLs in the gene expression prediction phase of TWAS, potentially enhancing the accuracy of TWAS. Other molecular Quantitative Trait Loci (QTL) play a crucial role in unraveling the genetic basis of diseases, including sQTL (splicing QTL) [57], apaQTL (alternative polyadenylation QTL) [58], mQTL (methylation QTL) [59], cQTL (chromatin QTL)[60], hQTL (histone QTL)[61] and more[62], [63], [64]. However, despite the association of thousands of SNPs with these molecular events, it is essential to accurately determine the causal SNPs. Without this, the analysis of these QTLs may hold limited significance, as a substantial portion of them might not be causal. Furthermore, experimentally validating thousands of SNPs can be an arduous task for biologists. The algorithmic approach of CausalEQTL shows promise for future applications in the precise detection of these various molecular QTLs.

## CRediT authorship contribution statement

**Guishen Wang:** Conceptualization, Methodology, Writing - original draft, Writing - review & editing, Funding acquisition. **Hangchen Zhang:** Software, Investigation, Data Curation, Visualization, Writing - original draft. **Mengting Shao:** Validation, Visualization, Writing - original draft, Writing - review & editing. **Min Tian:** Software, Investigation, Writing - Review & Editing. **Hui Feng:** Formal analysis, Writing - review & editing. **Qiaoling Li:** Supervision, Writing - review & editing. **Chen Cao:** Conceptualization, Methodology, Software, Supervision, Writing - original draft, Writing - review & editing, Funding acquisition.

## Declaration of Competing Interest

The authors declare that they have no known competing financial interests or personal relationships that could have appeared to influence the work reported in this paper.

## Data Availability

The Genotype-Tissue Expression (GTEx) Project was supported by the Common Fund of the Office of the Director of the National Institutes of Health, and by NCI, NHGRI, NHLBI, NIDA, NIMH, and NINDS. The data used for the analyses described in this manuscript were obtained from the GTEx Portal and the GTEx individual-level genotypes were obtained from the dbGaP (dbGaP Study Accession: phs000424.v9.p2), https://www.ncbi.nlm.nih.gov/projects/gap/cgibin/study.cgi?study_id= phs000424.v9.p2. PLINK, https://www.cog-genomics.org/plink/. The source code and related data are available at https://github.com/zhc-moushang/CausalEQTL.
