# Supplementary material for: Optimal variable identification for accurate detection of causal expression Quantitative Trait Loci with applications in heart-related diseases
Source: Comput Struct Biotechnol J. 2024 Jun 3;23:2478–86. doi: 10.1016/j.csbj.2024.05.050 (PMC11215961; doi:10.1016/j.csbj.2024.05.050)
Supplement: Supplementary file 1 — Supplementary material [file mmc1.docx]

**Optimal Variable Identification for Accurate Detection of Causal Expression Quantitative Trait Loci with Applications in Heart-Related Diseases**

**Supplementary Material**

Guishen Wang^a,1^, Hangchen Zhang^a,1^, Mengting Shao^b,1^, Min Tian^b^, Hui Feng^a^, Qiaoling Li^c,*^, Chen Cao^b,*^

^a^ College of Computer Science and Engineering, Changchun University of Technology, Changchun, 130012, China

^b^ Key Laboratory for Bio-Electromagnetic Environment and Advanced Medical Theranostics, School of Biomedical Engineering and Informatics, Nanjing Medical University, Nanjing, 211166, China

^c^ Department of Cardiology, Affiliated Drum Tower Hospital, Medical School of Nanjing University, Nanjing, 210008, China

^*^ Corresponding author.

^**^ Corresponding author at: Key Laboratory for Bio-Electromagnetic Environment and Advanced Medical Theranostics, School of Biomedical Engineering and Informatics, Nanjing Medical University, 211166, Nanjing, China

E-mail addresses: lqldoctor@126.com (Q. Li) and chen.cao@ucalgary.ca (C. Cao).

^1^ These authors contributed equally to this work: Guishen Wang, Hangchen Zhang, Mengting Shao.

**REAL DATA COMPARISON RESULTS**

**Table S1. Whole blood tissue results**

| Methods | Selected number | Correct number | Accuracy |
| --- | --- | --- | --- |
| CausalEQTL | 14092 | 6899 | 48.9% |
| CaVEMaN | 7816 | 5436 | 69.5% |
| LASSO | 37062 | 16255 | 43.8% |
| Elastic Net | 50075 | 23421 | 46.7% |
| DAP-G | 181614 | 88458 | 48.7% |
| TensorQTL | 70708 | 63899 | 90.3% |

**Table S2. Muscle tissue results**

| Methods | Selected number | Correct number | Accuracy |
| --- | --- | --- | --- |
| CausalEQTL | 15513 | 7739 | 49.8% |
| CaVEMaN | 8831 | 6360 | 72.0% |
| LASSO | 37974 | 17677 | 46.5% |
| Elastic Net | 52312 | 26176 | 50.0% |
| DAP-G | 207898 | 99985 | 48.0% |
| TensorQTL | 75919 | 70227 | 92.5% |

We compared the results of different methods in whole blood tissue (Table S1) and muscle tissue (Table S2), which are the two tissues with the largest sample sizes. Whole blood tissue contained 670 samples, while muscle tissue contained 571 samples. After preprocessing, there were 6070 genes in whole blood tissue and 6594 genes in muscle tissue. In Table S1, the statistical methods included CausalEQTL, LASSO and Elastic Net with accuracies of 48.9%, 43.8% and 46.7%, respectively. In our simulation, optimalLambda is set to 0.001.

In Table S2, we observe that the data does not change much, but the results of Elastic Net fluctuate greatly. We speculate that this is due to changes in the sample sizes.

**PARAMETER SELECTION AND OPTIMIZATION**

**Table S3. Parameter list**

| Parameter | Value |
| --- | --- |
| nFolds | 5 |
| seed | 2 |
| nGamma | 10 |
| gammaMin | 0.001 |
| gammaMax | 1.0 |
| maxSuppSize | 10 |
| intercept | FALSE |
| algorithm | CDPSI |
| optimalLambda | 0.001 |

Details of the parameters we use are in Table S3. The nFolds represents the number of cross-validation folds. We use the officially recommended 5-fold cross-validation. The seed is a random seed to ensure that the results can be reproduced.The nGamma represents the number of Gamma values to select. This parameter significantly increases computation time. We chose 10 gamma values as we are utilizing cluster computing. gammaMin and gammaMax set the range of Gamma values. maxSuppSize limits the number of non-zero elements in the coefficient matrix. In our calculations, for example, 14,092 results were selected from 6,070 genes in whole blood tissue. On average, 2 to 3 results were selected for each gene, which is far less than the maximum value of 10 we set. Additionally, this parameter has minimal impact on the results. intercept is set to FALSE because we do not need to use an intercept in our work. There are two algorithm options CDPSI ,CD. CDPSI performs local combinatorial search on top of CD and typically achieves higher quality solutions (at the expense of increased running time). We use cluster computing, so we choose CDPSI to get better results. OptimalLambda has a certain impact on the number of selections. If OptimalLambda is too small, the number of selections increases, and the number of correct selections also increases. However, the accuracy is lower. For example, in muscle tissue, when OptimalLambda is 0.0005, CausalEQTL selects 20,538 results, with an accuracy of 43.0%. When OptimalLambda is 0.005, CausalEQTL selects 5,826 results, with an accuracy of 77.4%. In order to balance accuracy rate and the number of selections, we set OptimalLambda to 0.001, resulting in an accuracy rate of 49.8% in muscle tissue.
